# The role of post-systolic strain and electrocardiographic changes during dobutamine stress echocardiography in enhancing detection of symptomatic coronary artery disease

**DOI:** 10.3389/fcvm.2025.1641044

**Published:** 2025-10-17

**Authors:** Aleksandra Zivanic, Ivan Stankovic, Ivona Vranic Jovanovic, Milos Panic, Milica Scepanovic, Aleksandra Maksimovic, Predrag Milicevic, Tijana Kalezic-Radmili, Aleksandar N. Neskovic

**Affiliations:** ^1^ Department of Cardiology, Clinical Hospital Center Zemun, Belgrade, Serbia; ^2^ Faculty of Medicine, University of Belgrade, Belgrade, Serbia

**Keywords:** dobutamine, stress echocardiography, coronary artery disease, microvascular dysfunction, electrocardiography

## Abstract

**Background:**

To enhance the diagnosis of coronary artery disease (CAD) during dobutamine stress echocardiography (DSE), subjective visual evaluation of left ventricular (LV) wall motion abnormalities may be complemented by analyzing myocardial deformation and electrocardiographic (ECG) changes.

**Aims:**

This study evaluates the post-systolic strain index (PSI) measured during the recovery phase of DSE for detecting obstructive CAD and explores its relationship with wall motion abnormalities and ECG changes during DSE in patients with anginal symptoms.

**Methods:**

We retrospectively analyzed data from 72 patients who underwent both DSE and coronary angiography. We compared visual interpretation of DSE at peak stress, ECG abnormalities during DSE, and PSI during the recovery phase with obstructive CAD.

**Results:**

LV wall motion abnormalities induced by dobutamine were independently associated with obstructive CAD [odds ratio (OR) 8.58, 95% confidence interval (CI) 2.67–27.50, *p* < 0.011], diagnosed in 44% of patients. Significant ECG changes during DSE correlated with obstructive CAD (OR 4.41, 95% CI 1.41–13.81, *p* = 0.011). PSI during recovery did not correlate with DSE-induced wall motion abnormalities (OR 1.45, 95% CI 0.49–4.24, *p* = 0.497) or obstructive CAD (OR 1.00, 95% CI 0.342–2.926, *p* = 1.00), but was associated with pathological ECG changes (OR 5.51, 95% CI 1.05–28.99, *p* = 0.044).

**Conclusions:**

PSI measured during the recovery phase of DSE is not associated with DSE-induced wall motion abnormalities and obstructive CAD in patients with anginal symptoms. However, PSI may be related to ECG changes and could potentially reflect subtle, stress-induced myocardial dysfunction, possibly involving coronary microvascular impairment.

## Introduction

Dobutamine stress echocardiography (DSE) is a widely used non-invasive test for detecting obstructive coronary artery disease (CAD) and demonstrates high accuracy when conducted by an experienced physician (1). However, the subjectivity of visual interpretation of left ventricular (LV) wall motion abnormalities is a limitation of the test (2), which could be improved by assessing myocardial deformation (i.e., post-systolic shortening, PSS) during high-dose DSE (3, 4). The assessment of myocardial deformation is typically performed with speckle tracking echocardiography, which can be technically challenging to perform at peak stress with a heart rate of 120–140/min. However, it has been shown that the detection of abnormal myocardial deformation patterns, such as PSS, during the recovery phase of the stress test (i.e., the ischaemic memory) can be a sign of transient ischaemia at peak stress (5). Therefore, quantifying DSE using speckle-tracking echocardiography during recovery seems promising due to easier image acquisition and potential for detecting ischaemic memory shortly after inducing ischaemia during a high-dose test (6, 7). However, it remains unclear if detecting ischaemic memory solely correlates with obstructive CAD or also signifies ischaemia from microvascular dysfunction. The 12-lead electrocardiogram (ECG) is an integral part of DSE, yet its role in the detection of myocardial ischaemia and obstructive CAD is not yet fully understood. Recently, ST-segment depression on exercise ECG test has been shown to be highly specific for an underlying ischaemic substrate when microvascular dysfunction is taken into account (8). In addition, high-grade premature ventricular contractions (PVCs) occurring during the recovery phase of exercise ECG tests were associated with long-term risk of cardiovascular mortality (9). Also, in a recent meta-analysis evaluating the prognostic significance of stress test–induced arrhythmias during both exercise and pharmacological stress testing, frequent premature ventricular contractions (PVCs) were linked to higher mortality compared with infrequent PVCs (10). Prior studies have used varying thresholds to define frequent PVCs, including a cutoff of ≥5 PVCs per minute (11).

This observational retrospective study aimed to evaluate the associations between visual interpretation of DSE at peak stress, detection of ischemic memory through post-systolic strain during the recovery phase, ECG abnormalities during DSE, and obstructive CAD on coronary angiography, in patients with anginal symptom.

## Methods

This retrospective observational study included 72 patients with anginal symptoms who were referred to the Clinical Hospital Center Zemun for DSE due to suspected CAD and who also underwent coronary angiography due to positive DSE or persistent angina symptoms despite the negative test. Obstructive CAD was defined by invasive coronary angiography as >50% diameter stenosis of the left main coronary artery or >70% diameter stenosis in any other major epicardial coronary artery (12). The decision to perform invasive measurement of fractional flow reserve or non-hyperemic indices to assess the functional significance of intermediate lesions (diameter stenosis 30%–70%) was at the discretion of the interventional cardiologist. The study was approved by the Institutional ethics committee.

### DSE protocol

The DSE was conducted following a standard protocol that involves continuous infusion of dobutamine at incremental doses ranging from 5–40 mcg/kg/min with addition of up to 2 mg of atropine, as needed to achieve the target heart rate. Heart rate and blood pressure were monitored before, during, and after the test. The test was deemed non-conclusive if the target heart rate, defined as 85% of age-predicted maximal heart rate (220-age), was not achieved even with the maximum dose of atropine. The test was discontinued under the following criteria: achievement of the target heart rate, new LV asynergy at any test stage, occurrence of significant arrhythmia, systolic blood pressure increase to ≥220 mmHg, blood pressure decrease below baseline, or patient request due to unbearable chest pain or other discomfort. Patients were instructed to stop beta-blockers for two days prior to the test and not to use antianginal therapy on the test day. The echocardiographic assessment was performed using Vivid 7 and E9 machines (General Electric, Horton, Norway) with a 2.5 MHz transthoracic probe. Standard parasternal long-axis, short-axis at the level of the papillary muscles, and apical long-axis, 2-chamber, and 4-chamber views were acquired at rest, during low-dose dobutamine infusion (20 mcg/kg/min), at peak stress, and in the recovery phase with an average frame rate of 40–80 fps. Three consecutive cardiac cycles were recorded and digitally stored for offline analysis using the EchoPac workstation (version BT12; GE Healthcare). Regional LV systolic function was assessed immediately after the test using the “quad-view” mode on the ultrasound machine. A 17-segment model of the LV was used, with each segment graded on a four-degree scale (normokinesis, hypokinesis, dyskinesis, and akinesis). The test was considered positive for myocardial ischemia if new or worsening regional wall motion abnormalities were observed in at least two adjacent LV segments. All measurements adhered to international recommendations for chamber quantification (13).

A 12-lead ECG was recorded at baseline, at the end of each 5 min stage, and during the recovery phase. In this study, an ECG was considered pathological if it showed at least 1 mm of new horizontal or down-sloping ST-segment depression, ST-segment elevation or T-wave inversion in two contiguous leads or frequent PVCs (>5 per minute), as interpreted by the testing cardiologist (10, 11). An alternative definition of pathological ECG, excluding frequent PVCs, was also applied.

### Myocardial strain and two-dimensional speckle tracking analysis

**Figure 1:**
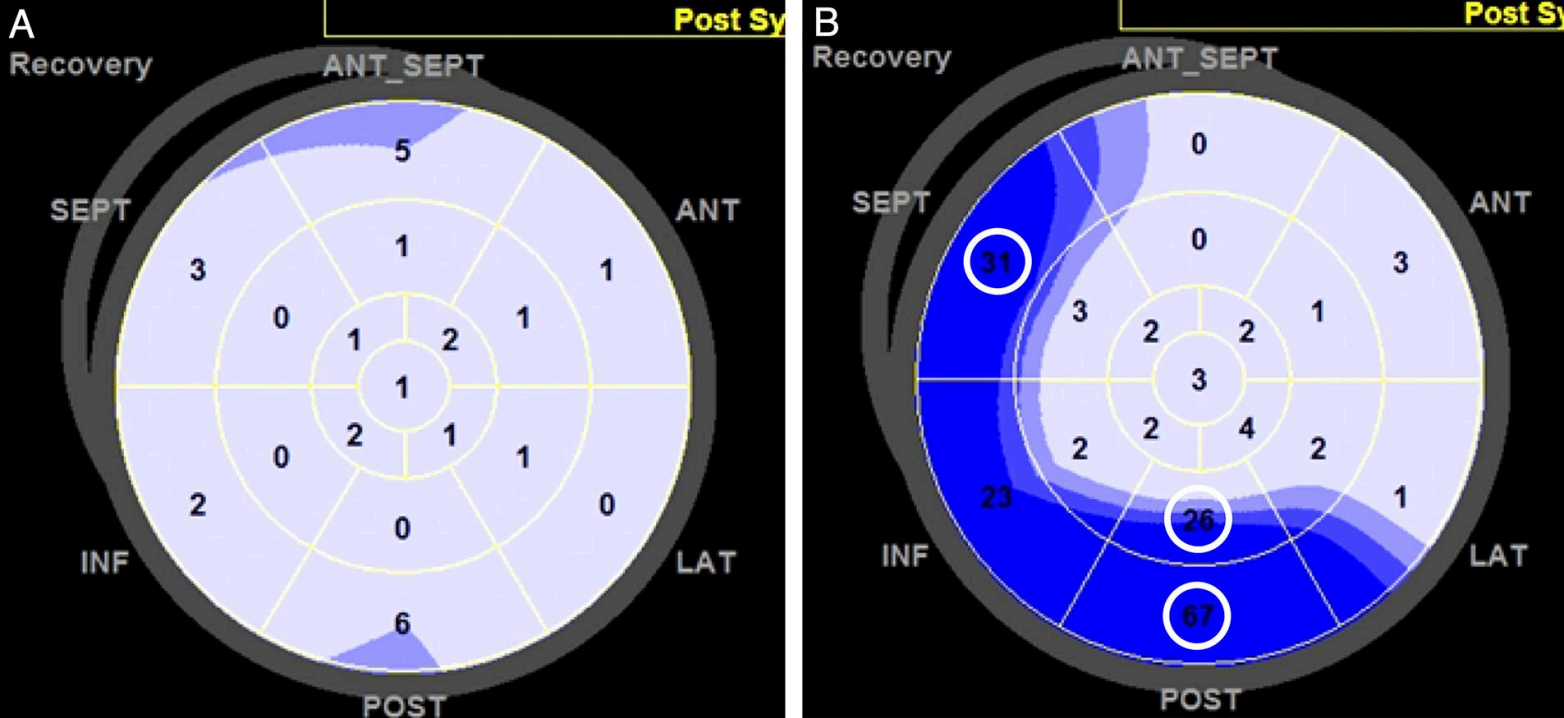
Assessment of the post-systolic index (PSI) during the recovery phase of dobutamine stress echocardiography (DSE). The PSI was automatically calculated for each left ventricular (LV) segment using software and displayed in a polar map as a percentage. PSI values were considered positive if two or more adjacent LV segments had PSI values exceeding the predefined cutoff of 25%. **(A)** Example of a patient with a negative PSI during the recovery phase, where all LV segments had low PSI values. **(B)** Example of a patient with a positive PSI, where several LV segments (circled) exhibited PSI values ≥25.

Myocardial deformation parameters were measured at three different points: before the test, during the low-dose test (20 mcg/kg/min), and during the recovery phase. Speckle-tracking analysis was performed using EchoPAC software (version BT12; GE Healthcare). Patients with more than two segments that could not be adequately tracked, even after manual adjustments, were excluded from the analysis. The software automatically generates longitudinal strain curves by tracking the endocardium and provides numerical values for peak systolic longitudinal strain and the post-systolic index (PSI) for each segment, as shown in the polar map view (Figure 1). Global longitudinal strain (GLS) was computed as the average of the peak systolic longitudinal strain values across all segments. The PSI was calculated by dividing the absolute difference between the amplitudes of systolic and post-systolic strain by the post-systolic strain. This value was then expressed as a percentage by multiplying by 100. Based on previous reports, ischemia was suggested if the PSI was ≥25% in two adjacent LV segments (14).

### Statistical analysis

Continuous data that are normally distributed are presented as means with standard deviations, while categorical data are summarized by frequencies and percentages. Comparisons of normally distributed continuous data between groups were performed using unpaired *t*-tests, and Fisher's exact test was used for categorical data. Normality was assessed using the Shapiro–Wilk and Kolmogorov–Smirnov tests. If these tests indicated a deviation from normality, the data were expressed as medians with interquartile ranges (IQR) and analyzed using the Mann–Whitney *U* test. Univariate and multivariate logistic regression analyses were conducted to examine associations between dobutamine-induced LV wall motion abnormalities, pathological ECG findings, PSI, and obstructive CAD. Intra- and interobserver variability of PSI ≥ 25% and GLS was assessed in 20 randomly selected patients using Kappa statistics and intraclass correlation coefficients (ICC), respectively. All statistical tests were two-tailed with a significance level set at *p* < 0.05. Statistical analyses were carried out using PASW Statistics version 23 (Chicago, IL, USA).

## Results

The characteristics of 72 patients included in the study are summarized in Table 1. Coronary angiography revealed obstructive CAD in 78% of patients with a positive DSE test, compared to 25% of those with a negative DSE test, resulting in a sensitivity of 78%, a specificity of 75%, and an overall accuracy of 76%. Among the 35 patients with a positive DSE test, 22 (63%) had 2 LV segments showing dobutamine-induced asynergy, while the remaining 37% had 3 ischemic segments. Additionally, of the 32 patients with obstructive CAD, 23 (72%) had single-vessel disease.

**Table 1:** Characteristics of the study population.

	All patients (*n* = 72)	Obstructive CAD (*n* = 32)	Nonobstructive CAD (*n* = 40)	*P*-value
Age, years	65 ± 9	66 ± 8	64 ± 9	0.537
Female sex, *n* (%)	44 (61)	16 (50)	28 (70)	0.095
Arterial hypertension, *n* (%)	64 (89)	28 (89)	36 (90)	1.000
Diabetes mellitus, *n* (%)	15 (21)	9 (28)	6 (15)	0.244
Dyslipidemia, *n* (%)	55 (76)	26 (81)	29 (73)	0.418
Family history of CAD, *n* (%)	35 (49)	18 (58)	17 (43)	0.236
Smoking, *n* (%)	31 (43)	18 (56)	13 (33)	0.057
Positive DSE, *n* (%)	35 (49)	25 (78)	10 (25)	<0.001
GLS at rest, %	18.1 ± 7.2	17.9 ± 7.4	18.2 ± 7.1	0.878
GLS during recovery, %	18.5 ± 4.0	17.5 ± 4.7	19.2 ± 3.2	0.084
PSI ≥ 25 during recovery, *n* (%)	18 (25)	8 (25%)	10 (25)	1.000
Pathologic ECG, *n* (%)	49 (68)	27 (84)	22 (55)	0.011
ST-segment depression, *n* (%)	44 (61)	24 (75)	20 (50)	0.051
T-wave inversion, *n* (%)	7 (10)	4 (13)	3 (8)	0.692
ST-segment elevation, *n* (%)	1 (1.4%)	1 (3%)	0	0.444
Frequent PVCs, *n* (%)	8 (11)	2 (6)	6 (15)	0.287

CAD, coronary artery disease; DSE, dobutamine stress echocardiography; ECG, electrocardiogram, GLS, global longitudinal strain, PSI, post-systolic indeks; PVC, premature ventricular contraction.

**Figure 2:**
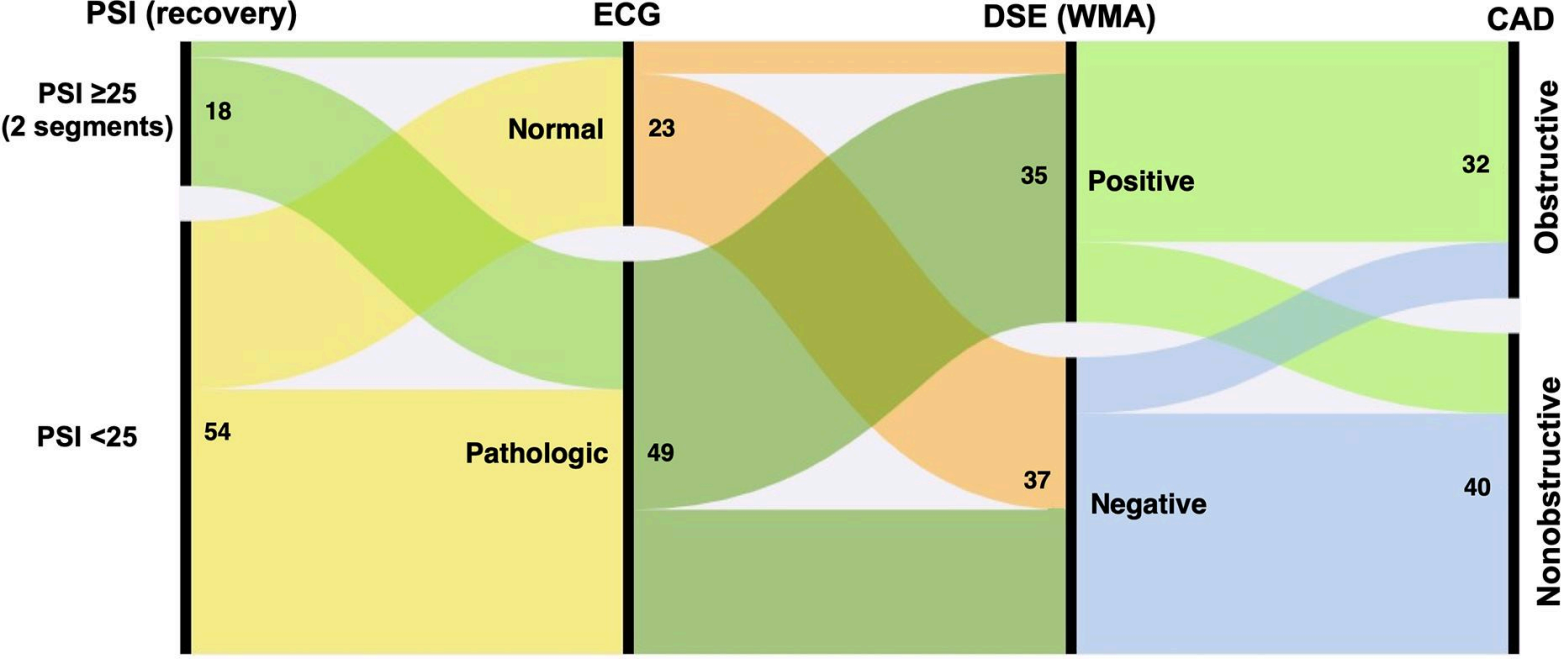
Alluvial plot illustrating associations between the post-systolic index (PSI), electrocardiogram (ECG), wall motion abnormalities (WMA), dobutamine stress echocardiography (DSE) and obstructive coronary artery desease (CAD). Numbers indicate the absolute number of patients in each category.

Patients with obstructive CAD were more likely to exhibit significant ECG changes during DSE than those without obstructive CAD. However, 55% of patients without obstructive CAD also showed pathological ECG changes during the DSE, primarily ST-segment depression. Figure 2 depicts the proportional relationships between ECG changes during DSE, dobutamine-induced wall motion abnormalities, PSI values during the recovery phase of DSE, and obstructive CAD.

The results of the univariate and multivariate regression analyses are shown in Table 2. In the univariate analysis, a positive DSE and pathological ECG during the test were associated with obstructive CAD, while PSI ≥ 25 during recovery was not. In the multivariate analysis, only a positive DSE remained independently associated with obstructive CAD.

**Table 2:** Univariate and multivariate regression analyses to identify parameters associated with obstructive CAD and pathologic electrocardiogram during dobutamine stress echocardiography.

	Univariate		Multivariate	
	OR (95% CI)	*P*-value	OR (95% CI)	*P*-value
Obstructive CAD				
Positive DSE (WMA)	10.714 (3.559–32.255)	<0.001	8.578 (2.669–27.503)	<0.001
Pathologic electrocardiogram	4.418 (1.414–13.809)	0.011	1.942 (0.516–7.305)	0.326
PSI ≥ 25 during recovery	1.000 (0.342–2.926)	1.000		
Positive DSE (WMA)				
PSI ≥ 25 during recovery	1.450 (0.496–4.238)	0.497		
Pathologic electrocardiogram during DSE				
PSI ≥ 25 during recovery	5.091 (1.061–24.433)	0.042	5.506 (1.045–28.998)	0.044
Positive DSE (WMA)	8.181 (2.404–27.893)	0.001	8.533 (2.416–30.143)	0.001

CAD, coronary artery desease; DSE, dobutamine stress echocardiography; PSI, post-systolic index, WMA, wall motion abnormalities.

Conversely, both a positive DSE and PSI ≥ 25 during the recovery phase were independently associated with pathological ECG changes during DSE. However, PSI ≥ 25 during recovery was not associated with positive DSE test, i.e., dobutamine-induced wall motion abnormalities.

Of note, when frequent PVCs were excluded from the definition of pathological ECG, PSI ≥ 25 during the recovery phase was no longer associated with pathological ECG changes during DSE (Supplementary Table S1).

**Figure 3:**
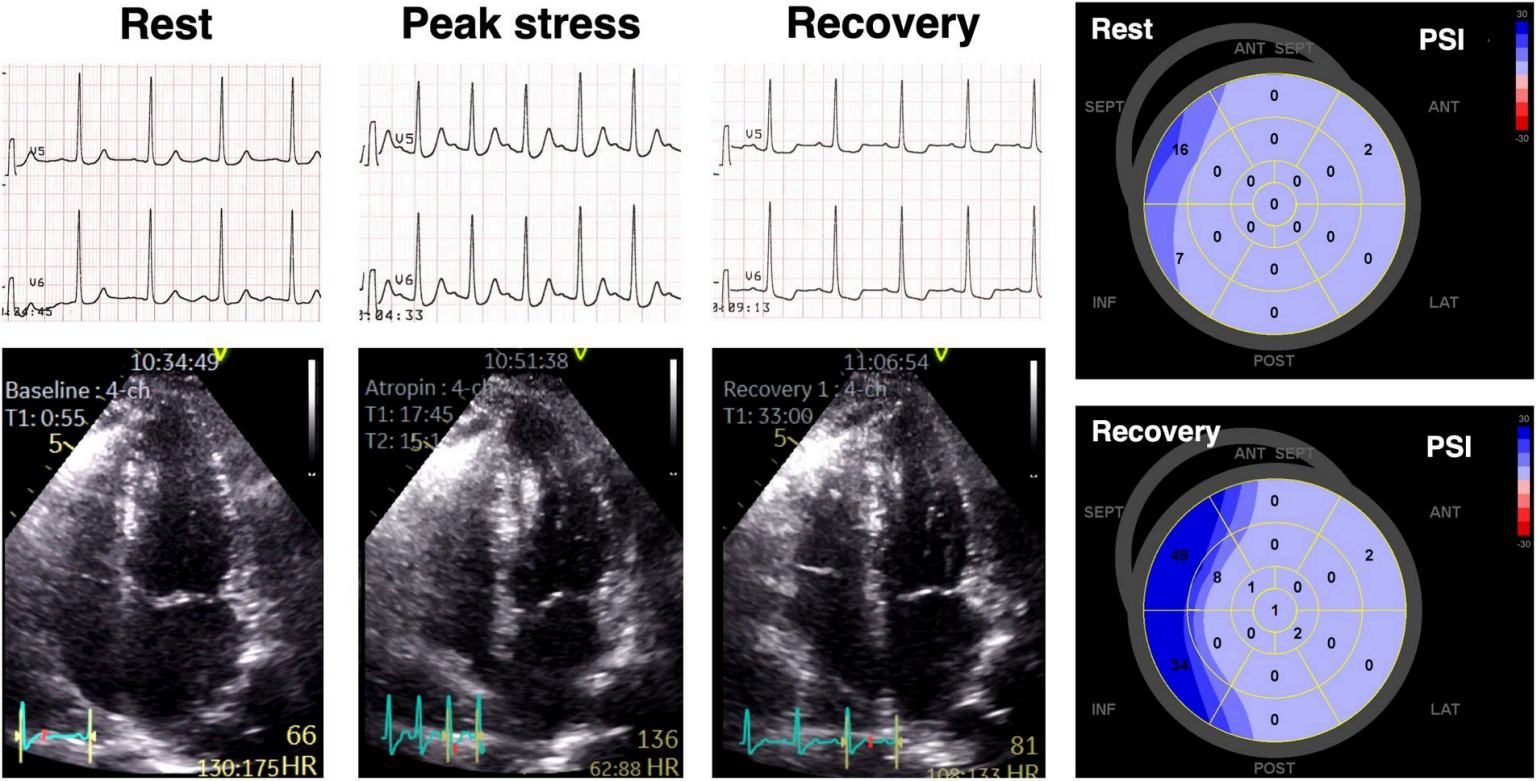
An example of a patient presenting with anginal symptoms and significant ST-segment depressions during dobutamine stress echocardiography (DSE), but with no obstructive coronary artery disease detected on invasive coronary angiography. Although there were no wall motion abnormalities observed during DSE, significant abnormalities in post-systolic myocardial deformation were evident, as indicated by elevated PSI values in multiple left ventricular segments.

Figure 3 illustrates an example of a patient with anginal symptoms and nonobstructive CAD who displayed significant ECG changes despite a negative DSE result.

For GLS, the intra-observer ICC was 0.999 (95% CI: 0.997–1.000) while the inter-observer ICC was 0.976 (95% CI: 0.927–0.994). For PSI ≥ 25, intra- and inter-observer assessments showed complete consistency, with 100% agreement.

## Discussion

In this study, PSI during the recovery phase of DSE was not associated with wall motion abnormalities observed during DSE or with obstructive CAD identified via coronary angiography. This finding challenges the assumption that PSI during the recovery phase enhances the diagnostic accuracy of DSE for detecting obstructive CAD (15). Despite this, our results suggest that PSI during recovery might still reflect subtle myocardial dysfunction, possibly associated with coronary microvascular disease. This aligns with the understanding that microvascular angina typically does not produce discernible wall motion abnormalities during DSE but is linked to symptoms and ECG changes during stress (16).

Quantifying myocardial deformation has been advocated as a more objective method for assessing regional LV systolic function during stress echocardiography, with previous research supporting its clinical utility (17). Although PSI during DSE has been demonstrated to be useful in identifying hemodynamically significant CAD (18–22), most studies have focused on myocardial strain analysis either at rest or at peak stress, with fewer examining the recovery phase.

However, a study by Uusitalo et al. found that assessing systolic or post-systolic strain using speckle-tracking echocardiography during early recovery after DSE provided additional diagnostic value for detecting obstructive CAD compared to visual analysis alone (15). The discrepancy between their findings and ours may be attributed to their use of myocardial perfusion positron emission tomography to document myocardial ischemia and obstructive CAD, a method not employed in our study. Additionally, the high percentage of a single-vessel CAD and the small zone of LV asynergy at peak stress in our cohort may have also contributed to the observed differences.

It is important to note that PSI, while sensitive, is not highly specific for detecting hemodynamically significant coronary artery stenoses (22–24). For instance, Lanza et al. observed that myocardial perfusion abnormalities detected by cardiac magnetic resonance during pharmacological stress testing correlated with reduced coronary microvascular vasodilation (25). Their findings, consistent with ours, highlighted pathological ECG changes, including ST-segment depression, in many patients. Similarly, Bhat et al. (26) investigated patients with positive treadmill tests and normal coronary angiograms, revealing impaired longitudinal strain assessed via speckle-tracking during DSE. However, unlike our study, which evaluated myocardial deformation during the recovery phase, Bhat et al. focused on strain measurements at rest and peak stress.

A recent study by Sinha et al. demonstrated that ischemic ECG changes during exercise testing were highly specific for coronary microvascular dysfunction (8). This underscores the limitation of using obstructive CAD as the sole benchmark for evaluating the diagnostic accuracy of noninvasive imaging modalities in patients with suspected chronic coronary syndrome. As highlighted by our study and others, ST-segment depression during stress-induced angina may reflect myocardial ischemia from both obstructive epicardial CAD and microvascular dysfunction (27). Our study extends this understanding by suggesting that while microvascular dysfunction might not produce wall motion abnormalities during peak stress, it can still lead to subtle, subvisual mechanical dysfunction detectable through post-systolic strain analysis early after DSE.

### Study limitations

This study has some limitations. Firstly, its single-center, retrospective design may limit the generalizability of the findings. All tests were performed as part of routine clinical practice, and the decision to pursue additional diagnostic testing was made by the referring cardiologists. Consequently, many patients underwent coronary angiography following DSE without utilizing other diagnostic methods that could offer higher sensitivity and specificity for detecting obstructive CAD. Additionally, the diagnosis of microvascular dysfunction in our study is provisional, based on the presence of anginal symptoms, DSE-induced ECG changes, and the exclusion of obstructive CAD through coronary angiography. Therefore, our observation of an association between PSI, ECG changes, and microvascular dysfunction should be regarded as hypothesis-generating only, since microvascular dysfunction was not directly assessed (e.g., by coronary flow reserve or positron emission tomography).

Future studies incorporating a multi-center design and a broader range of diagnostic modalities are warranted to validate these findings and better elucidate the role of post-systolic strain in assessing mechanical abnormalities in coronary microvascular dysfunction.

## Conclusions

In patients with anginal symptoms, PSI measured during the recovery phase of DSE is not associated with DSE-induced wall motion abnormalities and obstructive CAD. However, PSI may be related to ECG changes and could potentially reflect subtle, stress-induced myocardial dysfunction, possibly involving coronary microvascular impairment.

## Data Availability

The raw data supporting the conclusions of this article will be made available by the authors, without undue reservation.
