# Supplementary material for: The role of post-systolic strain and electrocardiographic changes during dobutamine stress echocardiography in enhancing detection of symptomatic coronary artery disease
Source: Front Cardiovasc Med. 2025 Oct 17;12:1641044. doi: 10.3389/fcvm.2025.1641044 (PMC12575320; doi:10.3389/fcvm.2025.1641044)
Supplement: Supplementary file 1 [file Table1.pdf]

Supplementary Table 1. Univariate and multivariate regression analyses to identify parameters associated with obstructive CAD and pathologic electrocardiogram (excluding frequent premature ventricular complexes) during dobutamine stress echocardiography

	**Univariate**		**Multivariate**	
	**OR [95% CI]**	**P-value**	**OR [95% CI]**	**P-value**
**Obstructive CAD**				
Positive DSE (WMA)	10.714 [3.559-32.255]	<0.001	7,945 [2.510-25.150]	<0.001
Pathologic electrocardiogram	5.400 [1.731-16.848]	0.004	2,721 [0.751-9,854]	0.127
PSI≥25 during recovery	1.000 [0.342-2.926]	1.000		
**Positive DSE (WMA)**				
PSI≥25 during recovery	1.450 [0.496-4.238]	0.497		
**Pathologic electrocardiogram during DSE**				
PSI≥25 during recovery	3,437 [0,888--13.303]	0.074	3,534 [0,830-15,049)	0.088
Positive DSE (WMA)	7,059 [2.243-22.214]	0.001	7,149 [2,214-23.081]	0.001

CAD - coronary artery desease; DSE - dobutamine stress echocardiography; PSI - post-systolic index, WMA – wall motion abnormalities.
